# An Agent-Based Model of Centralized Institutions, Social Network Technology, and Revolution

**DOI:** 10.1371/journal.pone.0080380

**Published:** 2013-11-21

**Authors:** Michael D. Makowsky, Jared Rubin

**Affiliations:** 1 Center for Advanced Modeling, Department of Emergency Medicine, Johns Hopkins University, Baltimore, Maryland, United States of America; 2 Department of Economics, Chapman University, Orange, California, United States of America; University of Warwick, United Kingdom

## Abstract

This paper sheds light on the general mechanisms underlying large-scale social and institutional change. We employ an agent-based model to test the impact of authority centralization and social network technology on preference falsification and institutional change. We find that preference falsification is increasing with centralization and decreasing with social network range. This leads to greater cascades of preference revelation and thus more institutional change in highly centralized societies and this effect is exacerbated at greater social network ranges. An empirical analysis confirms the connections that we find between institutional centralization, social radius, preference falsification, and institutional change.

## Introduction

Recent uprisings in Egypt, Libya, Tunisia, and other parts of the Arab world came quite unexpectedly to most observers. Although the seeds of discontent had been sown for decades in these countries, public anti-government displays barely existed. Such rapid changes in publicly displayed preferences are not a new phenomenon; precedents include the fall of Communism in the Eastern bloc, the end of apartheid in South Africa, and the civil rights movement [1], [2], [3], [4], [5], [6], [7]. For more on the mechanisms underlying rapid changes in publicly displayed preferences, see [6], [8], [9], [10], [11], [12], [13], [14], [15], [16], [17], [18], [19], [20], [21], [22], [23], [24], [25], [26].

We argue that economies containing two features – highly centralized power and widespread information and communication technology (ICT) – are conducive to massive and rapid preference revelation. We define power centralization as the ability of one actor to impose multiple sanctions on individuals. Examples include national and localized sanctions in autocracies, economic and religious sanctions in theocracies (such as Iran), or political and legal sanctions against dissidents (as in North Korea). Another example is provided by Goldstone [27], who in an article on the Arab Spring, notes that “Sultanistic governments” are particularly susceptible to revolutions. His definition of Sultanistic is very similar to our definition of centralization (italics ours): “Such governments arise when a national leader expands his personal power at the expense of formal institutions. Sultanistic dictators appeal to no ideology and have no purpose other than maintaining their personal authority. They may preserve some of the formal aspects of democracy—elections, political parties, a national assembly, or a constitution—but they rule above them *by installing compliant supporters in key positions* … Behind the scenes, such dictators generally amass great wealth, which they use to *buy the loyalty of supporters and punish opponents*. … Typically, the security forces are separated into several commands (army, air force, police, intelligence) —*each of which reports directly to the leader*. *The leader monopolizes contact between the commands*, between the military and civilians, and with foreign governments, *a practice that makes sultans essential for both coordinating the security forces and channeling foreign aid and investment*.” The ability of central authorities to impose sanctions on individuals, coupled with heterogeneous citizens whose true preferences are hidden, can calcify a society – leaving it stuck at sub-optimal equilibria despite changes to individual preferences.

Centralization can encourage individuals to publicly lie about their privately-held preferences (also known as “preference falsification” [4]) because those who transgress centralized authorities incur sanctions over numerous dimensions. For example, if one breaks religious dictates in Iran, they may suffer consequences in the afterlife as well as economic consequences in the present. Such societies are prone to cascades of preference revelation if preferences are inter-connected; that is, if individuals derive utility from conforming to the actions of others [4], [10], [13], [14]. A cascade can occur when a shock encourages some to reveal their privately-held preferences, which encourages others to do so, and so on. ICT helps facilitate this process; in order for the cascade mechanism to occur, people have to know how others are acting. ICT and preference falsification thus complement each other in the production of revolutionary activity; the former facilitates the transmission of the shock while the latter increases the magnitude of change that arises after a shock. These two phenomena reinforce each other; when both are present, revolutionary activity is most severe. This helps explain why highly centralized regimes (e.g., Libya, China, North Korea) frequently attempt to restrict information flows. Such regimes are precisely where revolutionary activity is most likely to occur (due to preference falsification); yet, when their citizens are weakly connected, the probability of a revolutionary cascade arising from a seemingly trivial shock decreases.

The popular notion that innovations in ICT are helping to facilitate the social and institutional changes we are witnessing in real time [28] is not without its detractors [29]. We argue that widespread ICT supports efforts to challenge authority by encouraging the public revelation of preferences. We model such actions in an agent-based framework to provide a better understanding of the mechanisms connecting political institutions, ICT, and revolutions. An empirical analysis using data from the World Values Survey, World Bank, and the Polity IV Project supports our primary argument that the likelihood of a massive institutional change is increasing in the degree of centralization and is exacerbated in highly centralized economies by widespread ICT.

## Methods

### Agent-Based Framework

The basic model linking institutional centralization and rapid, revolutionary change was proposed by Rubin [15]. It consists of heterogeneous agents who face costs when their actions differ from i) their internal preferences (or bliss points), ii) an endogenous social norm, iii) a central authority, and iv) a non-central authority. As an example, consider the motivations of some individuals taking part in the protests during the Arab Spring. There were certainly some people who desired moderate, non-violent protests against the government (this is their bliss point). Yet, the norm amongst their friends in their neighborhood was to take to the streets violently; hence, any non-violent protest carries a “social cost”. Of course, protesting violently carries other types of costs; namely jailing or worse by government forces (the central authority) and possibly spiritual sanctions by a local imam (a non-central authority).

Both authorities face costs from diverging from the citizenry, and the central authority can impose a cost on the non-central authority. The degree of centralization is increasing in the latter cost. We model centralization in this manner to highlight the idea that centralized power works through institutional conduits. For example, the religious hierarchy in Iran has power to impose political sanctions because the leading political authorities face significant costs from disobeying their dictates. Likewise, most autocrats impose multifarious sanctions through the military. In such a regime, the military is the “non-central” authority and the autocrat's degree of centralization hinges on how costly military authorities view choosing actions which defy the autocrat.

Rubin [15] suggests that citizens falsify their preferences in favor of the central authority in highly centralized regimes since they face multiple costs from transgression. Preference falsification occurs when people make public expressions different from their internal preferences. For example, preference falsification was rampant in Egypt, Tunisia, Syria, and Libya on the eve of the Arab Spring; there were clearly many people who detested their government, but they did not admit so publicly (at least, until the protests began). In other words, they publicly displayed preferences that were different from their internal beliefs. Preference falsification can unravel when a widespread shock alters the costs citizens face. If the shock is large enough, some citizens reveal their preferences, which alters the social norm, which itself encourages more citizens to reveal their preferences. A cascade can result, entailing a vastly different equilibrium of public expression.

A cascade of preference revelation is dependent on the means of social transmission. Social norms change only as people are made aware that the modal behavior in their social network is changing. Network structure has been shown to be relevant to the way in which behavior spreads through populations in game theoretic proofs [30], [31], [32], network theory [33], computational simulations [34], [35], [36], [37], and social experiments [38], [39].

We test the interactions between ICT, institutional centralization, and revolutionary activity with an agent-based model (ABM). Within our ABM we construct a population of autonomous, heterogeneous citizens whose rules-based decisions depend on, and in turn influence, the decisions of both their fellow citizens and the authorities that govern their artificial world. Agents occupy unique, randomly assigned spaces on a two-dimensional lattice, interacting with the members of their directed social network. We execute model simulations initializing the model and spinning it forward over discrete time steps. Macroscopic social patterns emerge from the interacting decisions made by agents over the course of a simulation [40], [41]. We conduct experiments exploring the sources of these patterns, running the model tens of thousands of times over a variety of model parameterizations.

### Model

The model is a repeated game played over *T* discrete steps in which *M* citizens engage in a game with a central authority (C) and a non-central authority (N). The central authority moves first, then the non-central authority, and finally the citizens. Within the set of citizens, the order of activation is randomized at each time step. The two authorities choose an action that maximizes their utility function, based in part on the mean citizen action from the previous time step.

**Figure 1:**
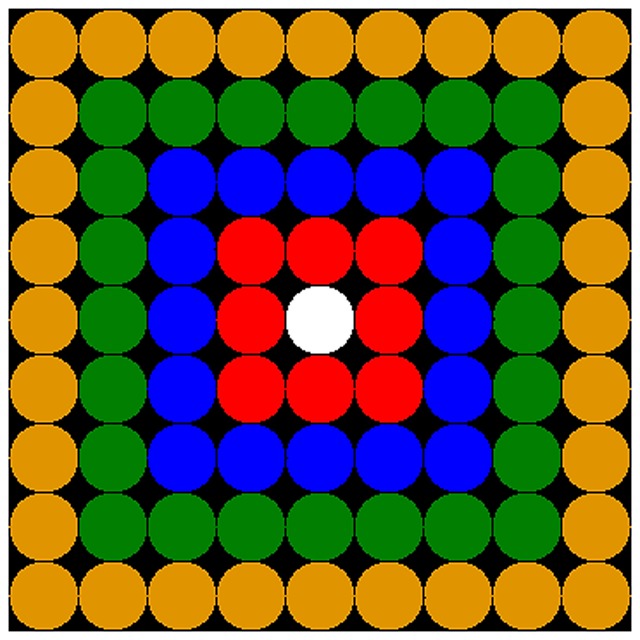
Citizen social radius on the lattice and network graph. r = 1(red agents), r = 2 (red, blue), r = 3 (red, blue,green), r = 4 (red, blue, grean, orange). The full lattice is 40 by 40 with 1600 agents.

The model is constructed on a 40 by 40 lattice with associated directed network graph. The model program was written using the MASON simulation Java library [42]. Agent social networks are subsets of selected agents from within their social radius *r*, which is the “Moore Neighborhood” of radius *r*. The Moore neighborhood is the square of surround cells on the lattice. The surveying agent is not included in his own neighborhood. The lattice is torus shaped and wraps at the edges, preventing edge effects. See Figure 1 below.

#### Step (t = 0) Model Initialization

The model creates and places agents randomly, one per lattice coordinate (x,y). Agents are heterogeneous across bliss point (*b_j_*) and are given random values from a normal distribution. All agents are initialized with actions equal to their bliss points. Citizens exist on a two-dimensional toroidal lattice. Their social network exists as a directed graph. Citizens actively form connections by choosing to connect to the *n* agents within radius *r* on the lattice whose actions *a_-i_* are closest to their own intrinsic bliss point *b_j_*. Networks are explored within the geographic context of a 2-D lattice. Results are qualitatively robust to the use of a Poisson distributed random network and the use of “small-world” networks with random rewiring of connections.

Table 1 indicates the order of action. The model analyzes situations in which the preferences of some citizens differ exogenously from those of the authorities, so actions could represent varying levels of freedom of speech, press, or religion, publicly expressed dissatisfaction with the government or religious authorities, or public opinion on social issues.

**Table 1:** Order of action (within step).

Central Authority( 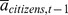 )
Non-central Authority( 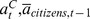 )
Citizens( 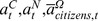 )†

†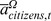 is the mean of the most recent actions taken by agents in the acting agent's social network. It includes a mixture of agents whose most recent actions were taken in the current step and agents whose most recent action was taken in the previous time step.

Citizens face three costs. Two of these costs are a function of the distance between the citizen's action (*a_j,t_*) and the actions of the two authorities, (*a^N^* and *a^C^*). These costs are increasing in the size of the violation and represent the costs (or punishments) associated with breaking a religious dictate, breaking a law, violating a political norm, and the like.

The third cost is a function of the distance between their action (*a_j,t_*) and the average action of other citizens within their social network (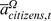). This norm is a property of the system that emerges from the interacting decisions of all of the agents. Each citizen j maximizes the following utility function in each period:
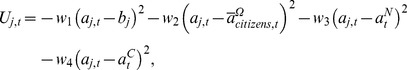
(1)


where *w_k_* is a weighting parameter greater than zero for 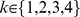. The utility function is concave, containing only a global maximum. This allows us to employ the relatively simple golden mean search optimization algorithm [43] in all agent utility maximization.

We define centralization as the weight (*γ*) that the non-central authority places on conforming to the central authority's dictates. The greater this weight is, the more influence the central authority has over the citizenry since they face multiple costs from transgressing the central authority. The central and non-central authorities have bliss points *b^C^* and *b^N^* and maximize the following utility functions in each period:
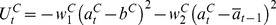
(2)

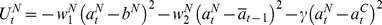
(3)


The fixed parameters employed in the ABM, which are constant across all model realizations, are reported in Table 2. Citizens first choose their social network and then choose their action. The choice of social network involves a survey of all of the citizens, θ*_i_*, within their social radius, *r*, such that 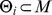. Citizens fill their network using a homophilous selection mechanism [33], [44], [45], ranking other citizens within their neighborhood and forming connections to the *n* other citizens, 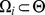, whose most recent actions are the closest to their personal bliss point.
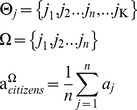
(4)


**Table 2:** Model parameters.

Parameter	Context/Related Function	Value
*M*	# Citizens	1600
*R*	Social radius	{1,2,3,4,5}
*γ*	Centralization	{0, 0.25, …4}
S	Shock fraction	{10%, 20%…100}
*Z*	Shock magnitude	3
N	Social network size	8
	All other utility function weights	0.5
t_shock_	Time step for social shock	*t* = 20
Bliss points	Central, Noncentral, mean citizen	{0.0, 0.5, 1.0}

* The lattice is a 40 by 40 torus with 100% agent density and no overlapping agents. Each combination of run parameters was simulated 50 times (n = 42,000).

Agent social networks are governed by two factors: the portion of the lattice over which an agent may search for agents to add to her social network and the total number of agents they choose to include in their network. The portion of the lattice they search is a function of their location on the lattice and the network radius parameter, *r*. In the two dimensional lattice of the model, the set θ*_i_* includes 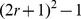 agents. The number of agents chosen in each social network, *n*, is a fixed exogenous parameter in the simulation experiments conducted.

### Step t = 20

At t = 20, a shock hits a portion of the citizenry. This shock increases the weight that they place on their bliss point vis-à-vis the social and institutional costs. Specifically, the shock multiples *w_1_* by a model parameter constant *Z*. The shock represents a change in the relative weights associated with acting in favor of one's internal preferences; whether this happens through a rise in one's weight on their intrinsic preference or a fall in the cost of sanctions yields the same results. Within the experiments conducted in this paper, *Z* is set to 3, tripling the weight agents place on their own intrinsic bliss point after the shock. Players continue to play the game as specified above until period t = 40, when the game ends. We chose to have the shock hit in period 20 because this ensures that a steady state has been reached. We are primarily concerned with how actions change from one equilibrium to another. Agents play the game for another 20 periods (until t = 40) so that a second steady state is reached.

## Results

The model was simulated over a range of parameterizations varying the levels of centralization, agent network radius, and the percentage of the population affected by shock. Each parameter combination was simulated 50 times, creating a total sample of 42,000 simulations.

### Preference Falsification

Centralized regimes employ numerous mechanisms to discourage the expression of anti-authority preferences. Commonly used tactics include detention without trial, partial jurisprudence, and beatings of dissenters. Such sanctions make it dangerous to express anti-government opinions even to seemingly close relations. In a state where most people publicly express favor for the government, it is difficult to discern who *actually* favors the government and who is *pretending* to favor the government. Where such mechanisms are present, many individuals engage in *preference falsification* [4], [46]. Mathematically, we define preference falsification as the difference between one's action (*a_j,t_*) and their bliss point (*b_j_*), since this indicates how much their expressed actions deviate from their internal preferences.

The primary path through which the centralization of sanctioning ability affects revolution is through preference falsification. In highly centralized societies, citizens are more likely to falsify their preferences since the sanctions from expressing anti-government views are greater. Social radius is also related to preference falsification. When individuals have fewer people with whom they can connect, they are less likely to run into people that have similar views, and thus the social norm that they follow is less likely to resemble their own preferences. Hence, ICT is important in part because it lets people know that others share similar views. This logic entails the following prediction:

#### Prediction 1

The degree of preference falsification is increasing in centralization and decreasing in social radius.

**Figure 2:**
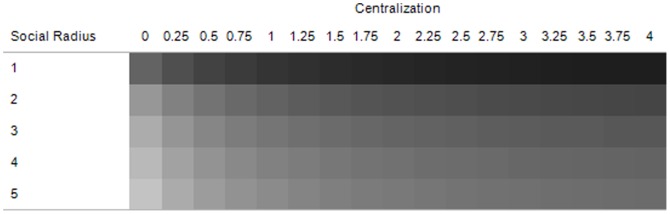
Preference falsification prior to the shock (t = 20).

Our model confirms this prediction. Figure 2 shows the degree of preference falsification over varying degrees of centralization and social radius, with darker areas indicating greater preference falsification. This figure reveals that preference falsification is indeed increasing in centralization and decreasing in social radius.

Consider next the determinants of preference revelation cascades. Where there is significant preference falsification, a shock encourages some citizens to express actions closer to their internal preferences, which in turn encourages more to act close to their bliss point, and so on. Hence, there should be a greater change in post-shock preference falsification where pre-shock preference falsification is greater. Moreover, there should also be greater post-shock changes in preference falsification where information travels faster, all else being equal. When information is known about the preferences of others, there is a lower cost of expressing one's internal preferences so long as others in one's social radius are doing the same.

The relationship between centralization and changes in preference falsification is therefore unambiguous. Greater centralization entails greater pre-shock preference falsification and thus a greater change in post-shock preference falsification. The following prediction follows from this logic:

#### Prediction 2

The change in preference falsification following a shock is increasing in centralization.

However, the relationship between social radius and changes in preference falsification is ambiguous. Greater social radii decrease pre-shock preference falsification (decreasing the likelihood of a cascade) but increase information flows (increasing the likelihood of a cascade). We call the former phenomenon the “preference falsification effect” and the latter phenomenon the “information effect”.

Perhaps more important is the interaction between centralization and social radius. At higher levels of centralization, the information effect is more important for precipitating cascades of preference revelation than it is at lower levels of centralization. Since preferences are falsified to a greater extent in highly centralized regimes, the feedback between information flows and preference revelation is stronger, suggests that there is a complementarity between the two in the production of cascades. In other words, at higher levels of centralization, the magnitude of the cascade should be increasing in social radius, with this effect being exacerbated as centralization increases. This logic entails the following prediction:

#### Prediction 3

At a sufficiently large level of centralization, the change in pre- and post-shock preference falsification is increasing in social radius. The degree to which it is increasing in social radius is increasing in centralization.

**Figure 3:**
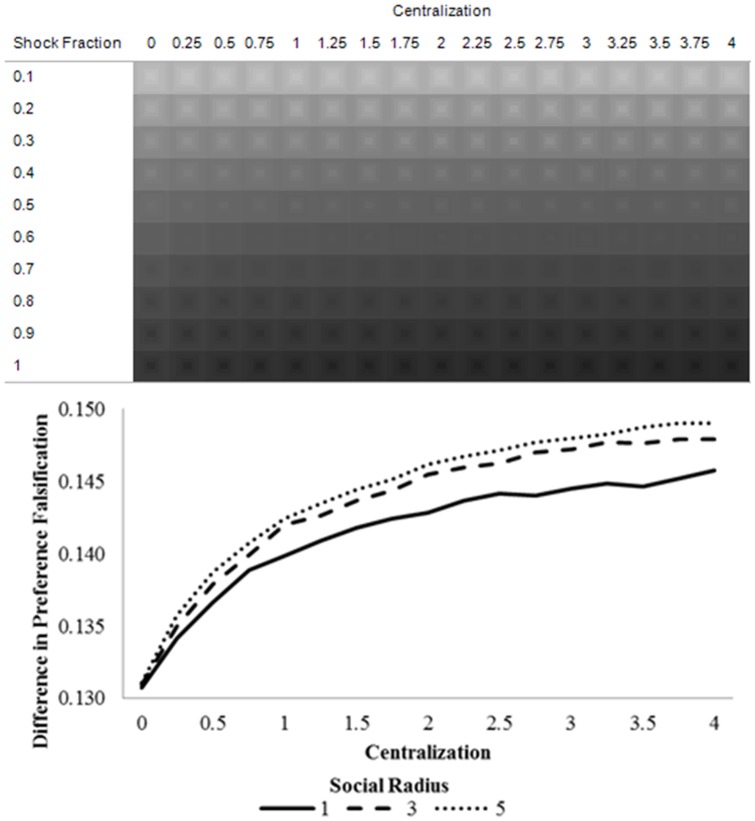
Average difference in preference falsification before (t = 20) and after (t = 40) the shock (upper region), and the average difference in preference falsification over centralization, pooled across shock fraction (lower region).

Predictions 2 and 3 are confirmed in Figure 3. The upper portion maps the difference in pre- and post-shock preference falsification over three dimensions: centralization, social radius, and the fraction of citizens affected by the shock. Lighter areas indicate less difference in preference falsification, darker areas indicate greater difference in preference falsification, and larger squares within each panel indicate a greater social radius. The lower portion of Figure 3 charts the change in preference falsification over centralization, pooling across shock fraction.

Several results are apparent in Figure 3. Figure 3 (lower) suggests that the change in preference falsification is increasing in centralization, with this effect being exacerbated at higher social radii. The former result is consistent with Prediction 2 and arises because pre-shock preference falsification is increasing in centralization. Moreover, the change in preference falsification diverges between social radii at higher levels of centralization, confirming Prediction 3, because the information effect complements preference falsification in the production of preference revelation cascades.

In Figure 3 (upper), it is apparent that the change in preference falsification is increasing with social radius when shocks are sufficiently small (≤60%). This is because the information effect is more important than the preference falsification effect when shocks are not directly transmitted to the vast majority of the population. It is in this circumstance that ICT is important for instigating preference revelation cascades since *transmission* is necessary for a cascade to emerge in the first place. However, when shocks are systemic (≥70%), enough people are affected by the shock that the preference revelation mechanism is not needed to transmit the shock and thus social radius matters less. In this case, the preference falsification effect dominates the information effect, since the widespread nature of the shock decreases the relative importance of the latter. This entails that the change in preference falsification is *decreasing* at large social radii when shocks are systemic.

In (Figure S1 in Appendix S1), we show how citizens' actions change before and after the shock. In particular, this Figure measures “protest”, which we define as the difference between the average citizens' action and the actions of the central authority. In other words, our protest measure indicates how far citizens are willing to openly transgress the central authority's dictates. Not surprisingly, these results are similar to those found for changes in preference falsification after the shock, so we do not elaborate on these results here.

How does the change in preference falsification manifest itself into institutional change? Under what conditions will institutional authorities respond to cascades of preference revelation with changes of their own? It is to these questions that we turn to next.

### Institutional Revolution

While the central authority influences outcomes through its ability to sanction deviant individual behavior, it is nonetheless beholden to the choices made by the citizen population. Since the central authority's utility is decreasing in the difference between its action and the mean action of the citizenry, it reacts to larger preference revelation cascades with larger changes to its action. In the previous section, we showed that cascades of preference revelation are more likely to occur when centralization is large, with this effect exacerbated as social radius increases. We thus expect the central authority's action to change after the shock in a similar manner.

The change in the central authority's action can be interpreted as the amount of institutional change that results from the shock. After all, this change is a measure of the degree to which institutional authorities change the laws and associated punishments in reaction to popular disapproval. We thus denote the degree to which the central authority changes its action in response to the shock as “institutional revolution.” In the real world, “institutional revolution” can be the result of the central authority pursuing new policies *or* the central authority being overthrown. For example, during the Arab Spring an “institutional revolution” occurred in Egypt and Libya, where the rulers were kicked out of power. Likewise, an institutional revolution also occurred in Bahrain, although the old regime stayed in power. In order to stay in power, it had to make numerous economic concessions, release political prisoners, and relieve many top officials of their duties. Mathematically, institutional revolution is defined as the difference between the central authorities' actions (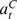) in the pre-shock steady state (period 20) and the post-shock steady state (period 40). Since we expect the change in the central authority's action to correspond to the change in preference falsification following the shock, the following two predictions are derived:

#### Prediction 4

The level of institutional revolution is increasing in centralization.

#### Prediction 5

The degree to which institutional revolution is increasing in social radius is increasing in centralization.

**Figure 4:**
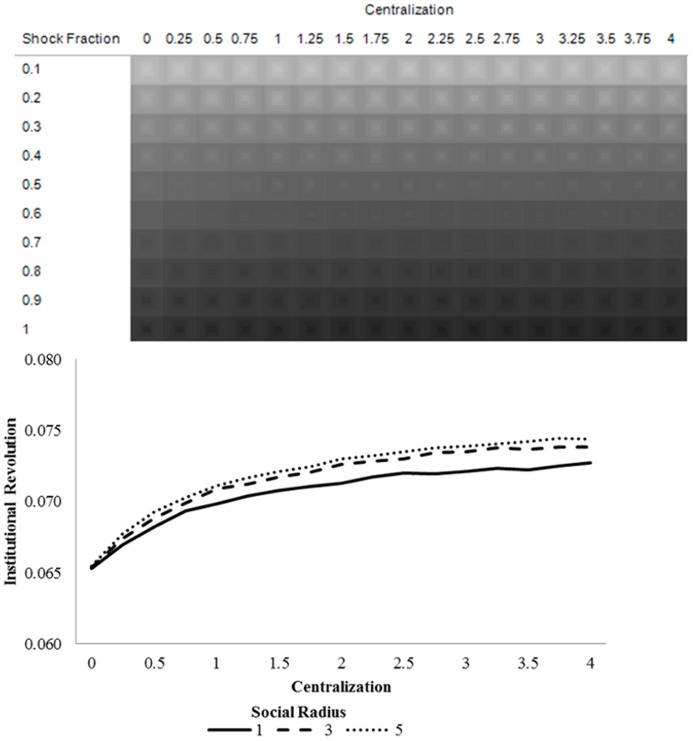
Average institutional revolution (Central action_40_ – Central action_20_) over centralization (upper region), and the average institutional revolution over centralization, pooled across shock fraction.

Figure 4 confirms Predictions 4 and 5. This figure maps institutional revolution over different parts of the parameter space.

These figures reveal the parameter combinations that are sufficient to tip the system towards a cascade that pushes the central authority towards significant change. Figure 4 (upper) suggests that a smaller shock is required for institutional change to arise in heavily centralized societies. This is because the overall level of institutional revolution is increasing in centralization, as indicated in Figure 4 (lower) and suggested by Prediction 4. Social radius exacerbates this effect in highly centralized regimes (Prediction 5), as large levels of pre-shock preference falsification combined with increased information flows reinforce the cascade of preference revelation and, in turn, institutional revolution.

**Figure 5:**
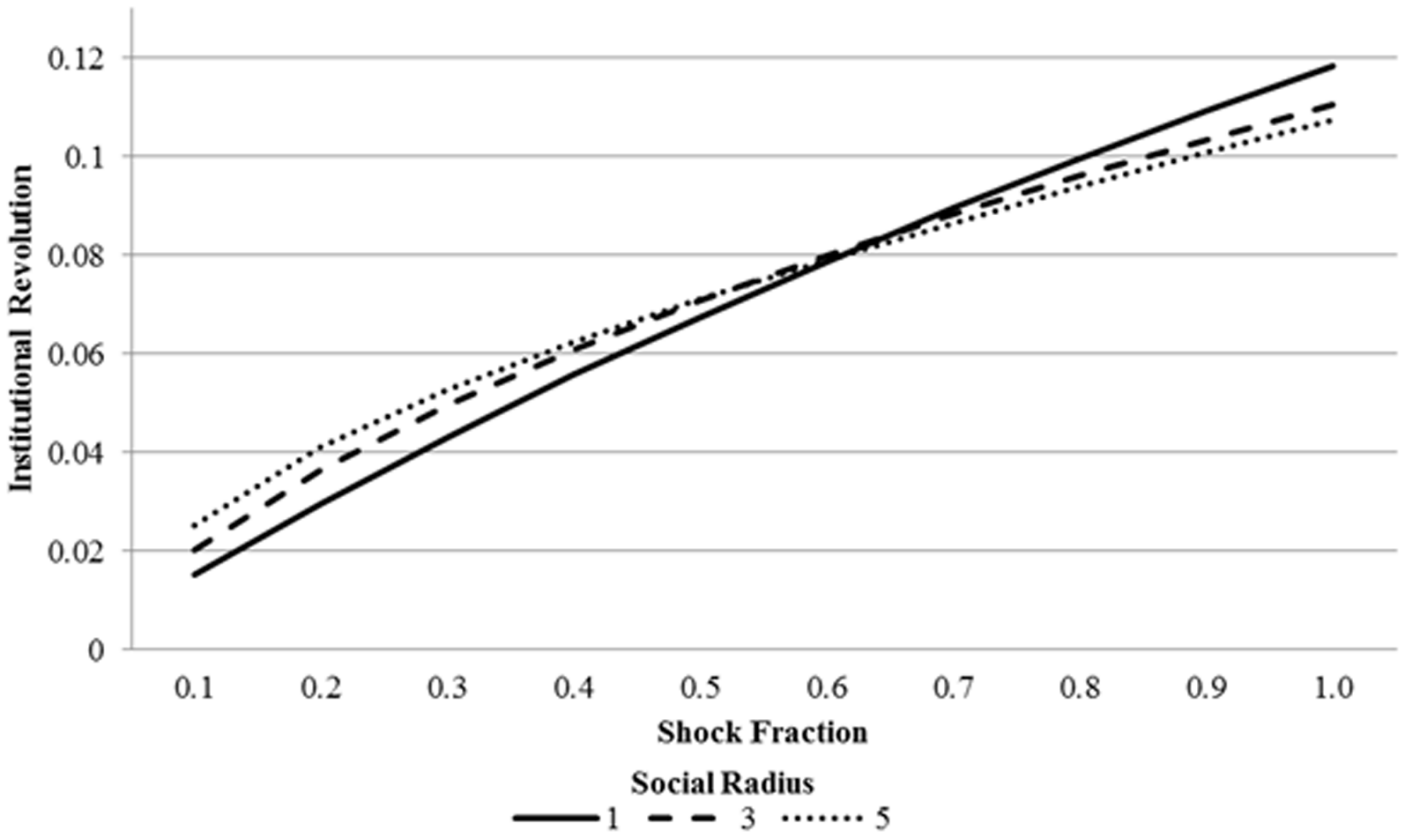
Average institutional revolution (Central action_40_ – Central action_20_) over shock fraction.

Figure 5 suggests that the amount of institutional revolution is monotonically increasing with shock size. However, as the social radius grows larger, the preference falsification effect and the information effect augment revolution in opposite ways. When the shock fraction is small, the information effect dominates, since the shock barely reaches much of the population and transmission is essential for it to have an effect. Hence, as Figure 5 suggests, the level of institutional revolution is increasing in social radius when the shock fraction is small (<0.6). However, when the shock fraction is large, a large social radius is less necessary to spread the shock (since most agents are directly affected by the shock), and the preference falsification effect dominates. That is, there is a larger amount of pre-shock preference falsification in low-radius societies, so a systemic shock causes larger preference revelation cascades. This entails that the level of institutional revolution is decreasing in social radius when centralization is sufficiently large (≥0.7).

## Empirical Analysis

The theory presented in the previous section makes multiple predictions relating institutional centralization, information and communication technology, preference falsification, and institutional change. Yet, supporting these predictions with empirical evidence can be an exercise in frustration. Survey data often provide the best information on individual preferences, but how can an investigator ascertain whether the preferences a subject revealed to their interviewer are the subject's true preferences? We attempt to work around this difficulty by moving from a micro to a macro context, and instead evaluate indicators of relative rates of preference falsification across populations rather than identify the true preferences of individuals.

To this end, we employ data from the World Values Survey, which has been conducted in numerous waves since 1981. Respondents are asked a variety of questions that relate to their opinions on various subjects, including personal and political values. Of interest to our study, respondents are asked about their views towards various protest activities, including 1) signing a petition, 2) joining a boycott, 3) attending a lawful/peaceful demonstration, 4) joining unofficial strikes, 5) occupying buildings or factories, 6) damaging property, or 7) engaging in personal violence. The survey asks both whether they *have* ever engaged in such activities and whether they *ever would*.

We exploit the respondents' revelation of protest activities that he or she would never engage in – their refusal to consider public protest as an option - as an indicator of preference falsification within the population. Specifically, we calculate *neverProtest_jt_* as the mean fraction of *K* protest activities (*q_h_*, for h = {1,2, …K}) that individuals *i,* from country *j,* during year *t,* claim they will never participate in:
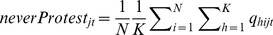
(5)


We begin the empirical analysis by analyzing the determinants of preference falsification. Prediction 1 and Figure 2 suggest that there is a negative (positive) relationship between social radius (centralization) and preference falsification. In other words, if the *neverProtest* variable is a good proxy for preference falsification, it should be negatively correlated with measures of social connectivity and positively correlated with measures of institutional centralization.

To address these hypotheses, we create proxies for social radius and institutional centralization. We proxy for the former with the number of Internet users per 100 people. This in part measures the degree to which people are connected to each other and how large their social networks are, so it should serve as a good proxy for social radius. We proxy for centralization with the constraint on the executive variable collected by the Polity IV Project: Political Regime Characteristics and Transitions, 1800–2010 [47]. This variable provides a nice proxy for the degree of institutional centralization, as we have defined it above, because it measures the degree to which political authorities can extend multifarious sanctions.

Returning to Prediction 1, our model suggests that preference falsification (proxied by *neverProtest*) should be increasing in centralization and decreasing in social radius. To test this prediction, we analyze the following equation using OLS:
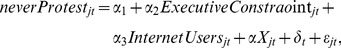
(6)


where *δ_t_* are time fixed effects and *X_jt_* is a vector of controls that might affect the average protest decision. These controls include log of GDP per capita, GDP growth, the unemployment rate, the percentage Muslim and Catholic (of those taking the survey), the log of survey respondents' average years of education, and the log of Battle related deaths (as reported in the Major Episodes of Political Violence and Conflict Regions, 1946–2008]). Summary statistics for all variables are listed in Table 3.

**Table 3:** Summary Statistics.

	N	Mean	Std. Dev.	Min	Max
neverProtest_jt_	149	0.561	0.166	0.084	0.920
Executive Constraint_jt_	146	2.265	1.618	1	7
Internet Users per 100	149	14.723	22.008	0	85.137
Regime Transition_jt_	149	0.349	0.478	0	1
Log GDP per capita (US 2000 $)	149	8.053	1.467	5.098	10.627
GDP Growth	149	4.033	4.507	−10.007	39.937
Unemployment Rate	145	0.148	0.101	0	0.531
% Muslim	149	0.151	0.291	0	0.993
% Catholic	149	0.283	0.312	0	0.944
Log Years of Education	137	1.451	0.217	0.805	1.878
Log of Battle Related Deaths	147	1	3	0	10.309

* The Executive Constraint variable is reversed from the Polity records – greater is executive constraints are reflected by lower scores; Log of Battle Deaths is actually log(1+Battle Related Deaths).

Table 4 reports the results of an OLS analysis of equation (6). All specifications include fixed effects for each wave of the World Value Survey and robust standard errors clustered by country. Column 1 reports results using only our independent variables of interest (without controls), where the Executive Constraint variable is transformed so that greater values indicate greater levels of centralization. Column two includes the control variables (*X_jt_*) listed above to account for economic, demographic, and political correlates of preference falsification. Consistent with Predictions 1 and Figure 2, both specifications suggest that there is a positive relationship between centralization (proxied by Constraint on the Executive variable) and preference falsification and a negative relationship between social radius (proxied by Internet users per 100) and preference falsification.

**Table 4:** Determinants of Preference Falsification.

Dependent Variable: *neverProtest_jt_*		
	(1)	(2)
Exec. Constraint	0.023***	0.020**
	(0.009)	(0.009)
Internet per 100	−0.004***	−0.003***
	(0.001)	(0.001)
Log GDP pc (US 2000 $)		−0.010
		(0.014)
GDP Growth		−0.004
		(0.004)
Unemployment		−0.099
		(0.157)
% Muslim		0.061
		(0.048)
% Catholic		0.047
		(0.053)
Log of Education		0.022
		(0.088)
Log of Battle Deaths		0.002
		(0.005)
Time Fixed Effects	Y	Y
Observations	146	128
R-squared	0.393	0.448
Number of Clusters	77	75

* OLS coefficients reported; all regressions include a constant term; standard errors clustered by country; the Executive Constraint variable is reversed from the Polity records (greater executive constraints are reflected by lower scores); *** p<0.01, ** p<0.05, * p<0.1.

More importantly, we are interested in how centralization and social radius affect institutional revolution. Predictions 4 and 5 suggest that centralization and social radius interact to affect the degree of institutional revolution that a society faces after a shock. In particular, these predictions suggest that institutional revolution is increasing in centralization, with this outcome being exacerbated as social radius increases. In order to test these predictions, we proxy for institutional revolution with the Polity IV “regime transition” indicator[47], denoted *RegimeTransition_jt_*. This variable identifies countries that experienced a significant institutional change during a wave of the World Value Survey. We model the determinants of *RegimeTransition_jt_* using the following two equations:
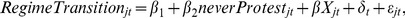
(7)

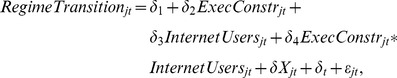
(8)


where *X_jt_* is the same vector of controls employed in equation (6). Equation (7) can be interpreted as the reduced form version of Equation (8), since the results reported in Table 4 suggest that preference falsification (*neverProtest_jt_*) is a function of centralization (*ExecConstr_jt_*) and social radius (*InternetUsers_jt_*). The interaction term in Equation (8) allows us to test whether the effect of centralization on institutional revolution is exacerbated at higher levels of social radius.

Table 5 presents results that confirm Predictions 4 and 5. This table reports the average marginal effects of probit regressions of the equations modeled in (7) and (8). Columns 1 and 2 indicate that preference falsification is correlated with institutional revolution, but only through centralization (and not through social radius). This result holds when controls are included in Columns 3 and 4. Prediction 5 indicates the effect of centralization on institutional revolution is *exacerbated* as social radius increases. To address this issue, column 5 reports the fully specified regression from equation (8), where the interaction between Executive Constraint and Internet users is included as an additional control. The coefficient on this interaction term is positive and statistically significant. This provides support for the model's prediction that social radius is indeed an important determinant of institutional revolution, but *primarily* through its interaction with institutional centralization.

**Table 5:** Regime Transition and Preference Falsification.

Dependent Variable: *RegimeTransition_jt_*					
	(1)	(2)	(3)	(4)	(5)
neverProtest	0.788***		0.793***		
	(0.225)		(0.296)		
Exec. Constraint		0.064***		0.060***	0.040*
		(0.021)		(0.020)	(0.021)
Internet per 100		−0.005		−0.002	−0.009
		(0.004)		(0.004)	(0.006)
Exec. Constraint * Internet					0.003*
					(0.002)
Log GDP pc (US 2000 $)			−0.058**	−0.037	−0.041
			(0.026)	(0.029)	(0.030)
GDP Growth			0.004	−0.014	−0.016
			(0.007)	(0.012)	(0.012)
Unemployment			0.405	0.233	0.240
			(0.394)	(0.356)	(0.356)
% Muslim			0.037	−0.036	−0.048
			(0.125)	(0.128)	(0.122)
% Catholic			−0.115	−0.072	−0.058
			(0.135)	(0.130)	(0.133)
Log of Education			−0.011	0.018	0.023
			(0.183)	(0.168)	(0.168)
Log of Battle Deaths			−0.004	−0.003	−0.002
			(0.013)	(0.011)	(0.011)
Time Fixed Effects	Y	Y	Y	Y	Y
Observations	155	154	131	135	135
Pseudo R-squared	79	80	77	78	78
Number of Clusters	0.140	0.188	0.244	0.245	0.262

* Average Marginal Effects of Probit regression reported; all regressions include a constant term; standard errors clustered by country; *** p<0.01, ** p<0.05, * p<0.1.

## Conclusions

Our results suggest that highly centralized regimes may seem tranquil but are highly susceptible to revolution, especially in large network-range economies. These results shed light on the institutional, technological, and social mechanisms facilitating the recent spread of revolutionary activity in the Arab world, the rapid decline of the Communist bloc, and numerous other instances where regime change occurred rapidly and unexpectedly in centralized societies.

An agent-based model highlights the role that information and communication technology play in triggering cascades of preference revelation in centralized societies. We show that network range reduces the minimum shock that is sufficient to effect institutional change, and this result is exacerbated as centralization increases. These results point towards a world where heavily centralized authorities are more likely to move towards the preferences of the general population in societies with increased access to modern ICT. At the same time, these results also reveal the incentive for central authorities to limit citizen access to ICT, including the internet and social networking.

## Supporting Information

Appendix S1 **Average difference in protest before (t = 20) and after (t = 40) the shock (upper region), and the average difference protest over centralization, pooled across shock fraction.**
(TIF)

## References

[1] Kuran T (1989) Sparks and prairie fires: A theory of unanticipated political revolution. Public Choice 61: 41–74.

[2] Kuran T (1991) Now Out of Never: The Element of Surprise in the East European Revolution of 1989. World Politics 44: 7–48.

[3] Kuran T (1991) The East European Revolution of 1989: Is it Surprising that We Were Surprised? The American Economic Review 81: 121–125.

[4] Kuran T (1995) Private Truths, Public Lies: The Social Consequences of Preference Falsification. Cambridge, MA: Harvard University Press.

[5] Kuran T (1995) The Inevitability of Future Revolutionary Surprises. American Journal of Sociology 100: 1528–1551.

[6] Lohmann S (1994) The Dynamics of Informational Cascades: The Monday Demonstrations in Leipzig, East Germany, 1989-91. World Politics 47: 42–101.

[7] Wright G (1999) “The Civil Rights Revolution as Economic History”. Journal of Economic History 59: 267–289.

[8] Banerjee AV (1992) A Simple Model of Herd Behavior. Quarterly Journal of Economics 107: 797–817.

[9] Bikhchandani S, Hirshleifer D, Welch I (1992) A Theory of Fads, Fashion, Custom, and Cultural Change as Information Cascades. Journal of Political Economy 100: 992–1026.

[10] Macy MW (1991) Chains of Cooperation: Threshold Effects in Collective Action. American Sociological Review 56: 730–747.

[11] Siegel DA (2009) Social Networks and Collective Action. American Journal of Political Science 53: 122–138.

[12] Schelling TC (1978) Micromotives and Macrobehavior. New York: W. W. Norton Company.

[13] Granovetter M (1978) Threshold Models of Collective Behavior. American Journal of Sociology 83: 1420–1443.

[14] Oliver PE, Marwell G, Teixeira R (1985) A Theory of Critical Mass. I. Interdependence, Heterogeneity and the Production of Collective Action. American Journal of Sociology 91: 522–556.

[15] Rubin J (2013) Centralized Institutions and Cascades. MPRA Paper 32364: University Library of Munich, Germany.

[16] Willer R, Kuwabara K, Macy Michael W (2009) The False Enforcement of Unpopular Norms. American Journal of Sociology 115: 451–490. 10.1086/599250 20614762

[17] Centola D, Willer R, Macy M (2005) The Emperor's Dilemma: A Computational Model of Self-Enforcing Norms. American Journal of Sociology 110: 1009–1040.

[18] Young HP (1993) The Evolution of Conventions. Econometrica 61: 57–84.

[19] Bicchieri C (2006) The grammar of society: the nature and dynamics of social norms. New York: Cambridge University Press. xvi, 260

[20] Ellis CJ, Fender J (2011) Information Cascades and Revolutionary Regime Transitions. Economic Journal 121: 763–792.

[21] Bernheim BD (1994) A Theory of Conformity. Journal of Political Economy 102: 841–877.

[22] Callander S (2007) Bandwagons and Momentum in Sequential Voting. Review of Economic Studies 74: 653–684.

[23] Kuran T, Sandholm WH (2008) Cultural Integration and Its Discontents. Review of Economic Studies 75: 201–228.

[24] Kuran T, Sunstein CR (1999) Availability Cascades and Risk Regulations. Stanford Law Review 51: 683–768.

[25] Watts DJ, Dodds PS (2007) Influentials, Networks, and Public Opinion Formation. Journal of Consumer Research 34: 441–458.

[26] Yin C-C (1998) Equilibria of Collective Action in Different Distributions of Protest Thresholds. Public Choice 97: 535–567.

[27] Goldstone JA (2011) Understanding the revolutions of 2011: weakness and resilience in Middle Eastern autocracies. Foreign Affairs 90..

[28] Shirky C (2011) The Political Power of Social Media. Technology, the Public

[29] Sphere, and Political Change. Foreign Affairs vol. 90: 28–41.

[30] Morozov E (2011) The net delusion: the dark side of internet freedom. New York: Public Affairs. xvii, 409 p.p.

[31] Morris S (2000) Contagion. The Review of Economic Studies 67: 57–78.

[32] Lee IH, Valentinyi Á (2000) Noisy Contagion without Mutation. The Review of Economic Studies 67: 47–56.

[33] Allen F, Gale D (2000) Financial Contagion. Journal of Political Economy 108: 1–33.

[34] Golub B, Jackson MO (2012) How homophily affects the speed of learning and best-response dynamics. Quarterly Journal of Economics 127: 1287–1338.

[35] Bonabeau E (2002) Agent-based modeling: Methods and techniques for simulating human systems. Proceedings of the National Academy of Sciences of the United States of America 99: 7280–7287. 12011407 10.1073/pnas.082080899 PMC128598

[36] Epstein JM (2002) Modeling civil violence: An agent-based computational approach. Proceedings of the National Academy of Sciences of the United States of America 99: 7243–7250. 11997450 10.1073/pnas.092080199 PMC128592

[37] Delre SA, Jager W, Bijmolt THA, Janssen MA (2007) Targeting and timing promotional activities: An agent-based model for the takeoff of new products. Journal of Business Research 60: 826–835.

[38] Cowan R, Jonard N (2004) Network structure and the diffusion of knowledge. Journal of Economic Dynamics and Control 28: 1557–1575.

[39] Centola D (2010) The Spread of Behavior in an Online Social Network Experiment. Science 329: 1194–1197. 20813952 10.1126/science.1185231

[40] Fowler JH, Christakis NA (2010) Cooperative behavior cascades in human social networks. Proceedings of the National Academy of Sciences 107: 5334–5338. 10.1073/pnas.0913149107 PMC2851803 20212120

[41] Epstein JM (2006) Generative social science: studies in agent-based computational modeling. Princeton: Princeton University Press. xx, 356 p.

[42] Epstein JM, Axtell R (1996) Growing artificial societies: social science from the bottom up. Washington, D.C.: Brookings Institution Press. xv, 208 p.

[43] Luke S, Cioffi-Revilla C, Panait L, Sullivan K, Balan G (2005) MASON: A Multiagent Simulation Environment. SIMULATION 81: 517.

[44] Press WH (2002) Numerical recipes in C++: the art of scientific computing. New York: Cambridge University Press. xxviii, 1002 p.

[45] Macy MW, Willer R (2002) From Factors to Actors: Computational Sociology and Agent-Based Modeling. Annual Review of Sociology 28: 143–166.

[46] McPherson M, Smith-Lovin L, Cook JM (2001) Birds of a Feather: Homophily in Social Networks. Annual Review of Sociology 27: 415–444.

[47] Kuran T (1987) Preference Falsification, Policy Continuity and Collective Conservatism. Economic Journal 97: 642–665.

[48] Marshall MG, Jaggers K (2012) Polity IV Project: Political Regime Characteristics and Transitions, 1800–2010. Societal-Systems Research Inc. and Colorado State University.

