# Supplementary figures and images for: An Agent-Based Model of Centralized Institutions, Social Network Technology, and Revolution
Source: PLoS One. 2013 Nov 21;8(11):e80380. doi: 10.1371/journal.pone.0080380 (PMC3837003; doi:10.1371/journal.pone.0080380)

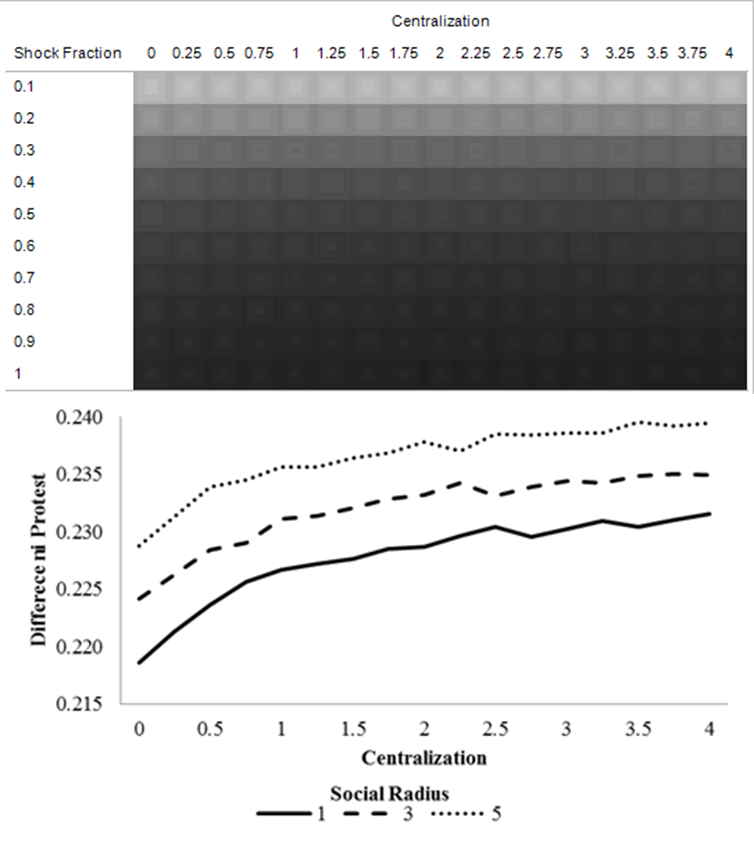

Supplement: Appendix S1 — Average difference in protest before (t = 20) and after (t = 40) the shock (upper region), and the average difference protest over centralization, pooled across shock fraction. (TIF) [file pone.0080380.s001.tif]
